# Epidemiological trends in burden of osteoarthritis in China: an analysis from 1990 to 2021 with forecasts for 2022–2050

**DOI:** 10.3389/fpubh.2025.1612596

**Published:** 2025-07-23

**Authors:** Xiaofei Cao, Ruixuan Zhu, Dan Liu, Yuanyuan Cheng, Yanmin Sun, Zhipeng Huang

**Affiliations:** ^1^Department of Orthopaedics, Tangdu Hospital, Fourth Military Medical University, Xi'an, China; ^2^The First Clinical Medical College, Xi’an Jiaotong University Health Science Center, Xi'an, Shaanxi, China

**Keywords:** osteoarthritis, disease burden, disability-adjusted life years, predictive analysis, disease burden analysis

## Abstract

**Objective:**

To analyze the epidemiological trends in the burden of osteoarthritis (OA) in China from 1990 to 2021 and predict trends to 2050.

**Methods:**

The publicly accessible modeling data derived from the Global Burden of Disease Study 2021 were employed. The annual percent change and the annual average percentage change were calculated to analyze the trend in the burden of OA. The Age-Period-Cohort (APC) model was used to analyze the age, period, and cohort effects on the incidence, prevalence, and DALYs of OA. The decomposition method was employed to analyze the changes in the burden of OA from 1990 to 2021 in China. Spearman’s correlation coefficients were used to assess correlations between the Age-Standardized Rates (ASRs) and the mean Age-Standardized prevalence from 1990 to 2021 in China. The Bayesian age-period cohort model was utilized to forecast the burden until 2050.

**Results:**

From 1990 to 2021, the age-standardized incidence rate (ASIR), age-standardized prevalence rate (ASPR), and age-standardized disability-adjusted life years rate (ASDR) of OA in China all exhibited an upward trend. In 2021, the ASIR and ASPR were 554.61 per 100,000 (95% UI: 486.85–619.54) and 7,030.66 per 100,000 (95% UI: 6,211.20–7,831.69), respectively. The ASDR was 244.79 per 100,000 (95% UI: 117.30–491.91). The age-standardized rates of OA were higher among females than among males in 2021. In different age groups, the incidence rate reached its peak in the 50–54 age group for both sexes. The decomposition analysis showed that aging, population growth, and epidemiological changes were driving an increase in the disease burden of osteoarthritis in China. The increase in the ASRs of OA in China is closely linked to the rise in obesity rates. The prediction results indicated that the ASIR first increased and then decreased, while the ASPR and ASDR showed an upward trend.

**Conclusion:**

In the future, the burden of OA in China is likely to continue to grow. Greater attention should be given to females aged 50–54 years, and rational prevention and control measures should be formulated.

## Highlights

- This study analyzed the disease burden and changing trends of OA in China between 1990 and 2021, and also made predictions for the period from 2022 to 2025.

## 1 Introduction

Osteoarthritis (OA) is a chronic ailment characterized by degeneration of articular cartilage and ranks among the most prevalent joint diseases globally (1, 2). Its symptoms primarily manifest as joint pain, stiffness and limited movement (3). Currently, there are no non-surgical interventions that can prevent, halt, or even slow the progression of OA, and available medications such as NSAIDs may be associated with a 50–100% increased risk of myocardial infarction or cardiovascular-related mortality compared to current clinical standards (4). With the progress of the disease, patients with advanced OA may suffer from joint deformity or even loss of mobility (5), thereby imposing an enormous burden on both patient health and the social economy (2, 6). Current clinical trial protocols still primarily rely on osteoarthritis definitions proposed in the mid-to-late-1900’s. The trials emphasizing symptom relief and imaging changes in joint space narrowing have yet to produce significant therapeutic advancements, making minimal progress in reducing disease burden (7). It is crucial for the public, healthcare providers, and policymakers to be aware of the heavy burden of OA.

OA has remained a major public health concern worldwide over the past decades. Despite significant progress in the study and treatment of OA over the past few decades (8, 9), other obstacles remain. Thus, prevention and early treatment are pivotal to mitigating the growing burden of OA. With the acceleration of the global aging process (10), urbanization (11), the rise of obesity (12) and lifestyle alterations such as lack of exercise, the high-risk population for OA is expanding (13). The escalating burden of OA has also been ascribed to the unequal distribution of medical resources (14), poor patient disease awareness, and a shortage of effective preventive strategies (15). The Global Burden of Disease 2021 (GBD 2021) study revealed that the global incidence rate of OA is increasing annually, rising from 391.86 per 100,000 in 1990 to 590.93 per 100,000 in 2021, representing an increase of 50.80%. In 2019, China was estimated to be the country with the highest prevalence of cases of OA (4). According to the seventh national census conducted in China in 2020, the population aged 60 and above reached 264.02 million, constituting 18.70% of the total population. The number of individuals aged 65 and above stood at 190.64 million, accounting for 13.50% of the total population (16). Over the past four decades, China has experienced a significant rise in overweight and obesity rates, coinciding with the country’s rapid economic growth, increasing globalization, and urbanization (17, 18). In 2021, there were approximately 133 million OA patients in China, accounting for 9.75% of the total number of all-cause diseases in China and 18.32% of the global total number of OA patients (19). In the near future, China is expected to face severe healthcare and economic challenges posed by osteoarthritis.

Current management strategies, such as pharmacological treatments, physical therapy, and surgery, primarily aim at symptom relief rather than a cure. Their efficacy varies among patients, and prolonged use may lead to potential adverse effects (20). Due to the lack of non-surgical treatment that can effectively prevent or treat OA, some patients with severe OA often need to receive joint replacement treatment (8). This implies that OA not only leads to physical dysfunction in patients but also imposes a substantial financial burden on families and society. Although joint replacement therapy has been widely adopted in clinical practice, the limited lifespan of the prosthesis after replacement (21) and the high cost of surgery (22) also exert a significant impact on the normal lives of patients. In summary, there is an urgent need for more effective diagnostic, treatment, and preventive methods. Consequently, early prevention and treatment of OA are the keys to alleviating the burden of OA (4, 15–23), and in-depth analysis of the changing trend of the OA disease burden, particularly the differences among various ages and sexes, holds great practical significance.

In recent years, only a few articles have described the burden of disease of OA in China based on the GBD 2019 database. In contrast, this study is based on the newly released GBD 2021. The number, crude rate of incidence, prevalence, and disability-adjusted life years (DALYs), along with age-standardized rates, were employed to analyze the epidemiological trends in burden of OA in China during the period from 1990 to 2021 and to predict the burden from 2022 to 2050. Through systematic data analysis, we aimed to disclose the prevalence characteristics of OA in different time periods, thereby providing a scientific basis for policymakers to better allocate medical resources and enhance the quality of life of patients. Additionally, this study would explore the prevention and treatment strategies for OA, promote the development of related research, and present new ideas and methods for addressing this increasingly severe public health challenge.

## 2 Materials and methods

### 2.1 Data source

The disease burden data of OA in China and globally utilized in this study were obtained from the GBD 2021 database.^1^ Data regarding the number, crude rate and age-standardized rate (ASR) of incidence, prevalence, DALYs during the period from 1990 to 2021 were selected. The GBD 2021 project is conducted by the Institute of Health Metrics and Evaluation (IHME). It employs standardized analysis methods to comprehensively evaluate 371 diseases (injuries) and 84 risk factors in 204 countries (regions) by age and sex. This analysis systematically adjusted epidemiological data to account for biases from varying data sources, definitions, and measurement methods. Complex statistical models, including MR-BRT and DisMod-MR 2.1, were employed to ensure internal consistency in estimates across regions, ages, sexes, and years to minimize the impact of heterogeneity on study results through standardization and calibration. The primary data sources in the GBD 2021 study for the hip and knee OA models were global, cross-sectional, population-based surveys, and state-level US insurance claims data, using ICD-10 codes M16 for hip and M17 for knee. GBD 2019 added two more OA sites: hand (M18) and other joints (M19), which were also included in GBD 2021 (24).

In the GBD framework, OA cases are defined as those with symptomatic osteoarthritis confirmed radiologically by Kellgren-Lawrence grading criteria of 2 to 4. The Kellgren-Lawrence grade 2 signifies the presence of a single defined osteophyte in the joint, grade 3 indicates multiple osteophytes and joint-space narrowing, grade 4 encompasses the criteria of grade 3, along with bone deformity, and the symptomatic osteoarthritis is defined by pain reported for at least 1 month within the past 12 months (25–27).

The study subjects of Adult Mean Age-Standardized Prevalence are adults aged between 25 and 125 years old with a BMI greater than 30. The data also originates from GBD 2021 database.

### 2.2 Statistical analysis

This study evaluated the burden of OA through indicators like incidence, prevalence, DALYs, and Age-Standardized Rates (ASRs), along with their respective 95% Uncertainty Intervals (UI). 14 age groups (ranging from 30–34. to 80–84, 85–89, 90–95, and ≥95) were selected for analysis. Data analysis and visualization were performed using R 4.3.3 and Joinpoint 4.9.1. The ASRs was calculated using the GBD world standard population age structure to eliminate differences in age composition between various regions or groups, making the data comparable.

The calculation formula for ASRs is:


$$\text{ASRs}=\frac{\sum_{i=1}^{A}\alpha_{i}\omega_{i}}{\sum_{i=1}^{A}\omega_{i}}\times 100,000$$

Where $\alpha_{i}$ represents the crude rates of the certain age group, $\omega_{i}$ represents the weight of people in the given age group in the standard population, A represents the number of age groups.

To assess the temporal trend of OA from 1990 to 2021, we conducted Joinpoint regression analysis to elucidate the changes in disease burden over time. This model employs segmented regression on a log-linear regression model to identify inflection points in the trend. We employed the Grid Search Method (GSM) to evaluate all potential join points, selecting the one with the lowest mean squared error (MSE) as the optimal inflection point. Subsequently, the optimal number of join points was determined using the Monte Carlo permutation test, with the range of 0 to 5 join points (28). The final model determined the Annual Percentage Change (APC), Average Annual Percentage Change (AAPC), and the associated 95% Confidence Intervals (CI), quantifying trend changes from 1990 to 2021. When APC is greater than 0, it indicates an annual increase; otherwise, it indicates an annual decrease. AAPC represents the overall trend change by weighting each segment’s APC based on the time span.

The calculation formulas for APC and AAPC are:


$${\mathrm{APC}}_{i}=\{\exp(b_{i})-1\}\times 100$$

$$\text{AAPC}=\{\exp(\frac{\sum \omega_{i}b_{i}}{\sum \omega_{i}})-1\}\times 100$$

Where $b_{i}$ represents the slope coefficient for the ith segment with *i* indexing the segments in the desired range of years, and $\omega_{i}$ represents the length of each segment in the range of years.

The Age-Period-Cohort (APC) model analyzes the impact of age, period, and cohort on health outcomes. The age effect pertains to the risk of outcomes at various ages. The period effect concerns the influence of temporal changes on outcomes across all age groups. The cohort effect involves changes in outcomes among individuals born in the same time period.

The log-linear regression model is expressed as:


$$\log(Y_{i})=\mu +\alpha *{\mathrm{age}}_{i}+\beta *{\text{period}}_{i}+\gamma *{\text{cohort}}_{i}+\varepsilon$$

Where $Y_{i}$ is the rate, α , β , and γ are the coefficients of age, period, and cohort, respectively, μ is the intercept and ε is the residual of model. The Intrinsic Estimator (IE) method integrated into APC model was used to get the net effects for three dimensions (29, 30). To run the APC model, we divided the data series into consecutive 5-year intervals from 1990 to 2019. Data from 2020 to 2021 data were not analyzed because they did not span a 5-year interval.

Additionally, we employed the Das Gupta decomposition method to analyze the changes in the burden of OA from 1990 to 2021 in China, which breaks down the changes into contributions from aging, population growth, and epidemiological shifts, providing clearer insights into how these factors have influenced trends over time (31).

Pearson’s or Spearman’s correlation coefficients were used to assess correlations between the ASRs and the mean Age-Standardized prevalence from 1990 to 2021 in China at the national level to identify potentially related factors.

This study employed the Bayesian Age-Period-Cohort (BAPC) model to predict future disease burdens due to its capacity to manage complex, high-dimensional, and sparse data frequently encountered in large-scale epidemiological studies like GBD 2021. The BAPC model builds on the traditional Generalized Linear Model (GLM) framework within a Bayesian context, allowing the dynamic integration of age, period, and cohort effects. These effects are assumed to evolve continuously over time and are smoothed using a second-order random walk, resulting in more accurate posterior probability predictions. A notable strength of the BAPC model is its use of the Integrated Nested Laplace Approximation (INLA) method for approximating the marginal posterior distribution. This approach effectively bypasses challenges such as mixing and convergence issues often associated with Markov Chain Monte Carlo techniques, while maintaining computational efficiency. The model’s flexibility and robustness in handling time series data make it particularly suitable for long-term disease burden predictions. Given its comprehensive coverage and ability to capture temporal trends, the BAPC model has been widely validated and applied in epidemiological research, especially in studies involving age-structured population data and complex cohort effects. This approach enables nuanced predictions of future disease burdens while considering the intricate interactions of age, period, and cohort effects (31, 32).

## 3 Results

### 3.1 Overall disease burden of OA from 1990 to 2021

In 2021, the incidence number of OA in China was 11,652,721.19 (95% UI: 10,207,638.21–13,107,929.18), and the number of patients was 152,848,105.93 (95% UI: 134,655,962.16–170,842,262.84). The age-standardized incidence rate (ASIR) and age-standardized prevalence rate (ASPR) were 554.61 per 100,000 (95% UI: 486.85–619.54) and 7,030.66 per 100,000 (95% UI: 6,211.20–7,831.69), respectively. In China, the DALYs attributed to OA was 5,327,389.98 person—years (95% UI: 2,541,781.20–10,678,674.95), and the age-standardized DALYs rate (ASDR) was 244.79 per 100,000 (95% UI: 117.30–491.91) (Table 1).

**Table 1:** Disease burden of different types of osteoarthritis in China from 1990 to 2021.

Variables	Incidence number (95% UI)	ASIR (/100,000) (95% UI)	Prevalence number (95% UI)	ASPR (/100,000) (95% UI)	DALYs (95% UI)	ASDR (/100,000) (95% UI)
1990	4,654,141.48 (4,075,192.05–5,212,885.65)	487.11 (428.13–543.75)	53,352,514.68 (46,603,087.10–59,685,781.45)	6,148.92 (5,417.29–6,855.85)	1,829,415.63 (880,107.77–3,682,519.91)	210.61 (101.91–423.86)
2021	11,652,721.19 (10,207,638.21–13,107,929.18)	554.61 (486.85–619.54)	152,848,105.93 (134,655,962.16–170,842,262.84)	7,030.66 (6,211.20–7,831.69)	5,327,389.98 (2,541,781.20–10,678,674.95)	244.79 (117.30–491.91)
Percentage change (%) (95% CI)	150.37 (142.75–157.10)	13.86 (11.96–16.05)	186.49 (179.62–192.93)	14.34 (12.13–16.45)	191.21 (183.62–198.69)	191.21 (183.62–198.69)

OA, osteoarthritis; ASIR, age-standardized incidence rate; ASPR, age-standardized prevalence rate; ASDR, age-standardized DALYs rate; DALYs, disability-adjusted life years.

**Figure 1:**
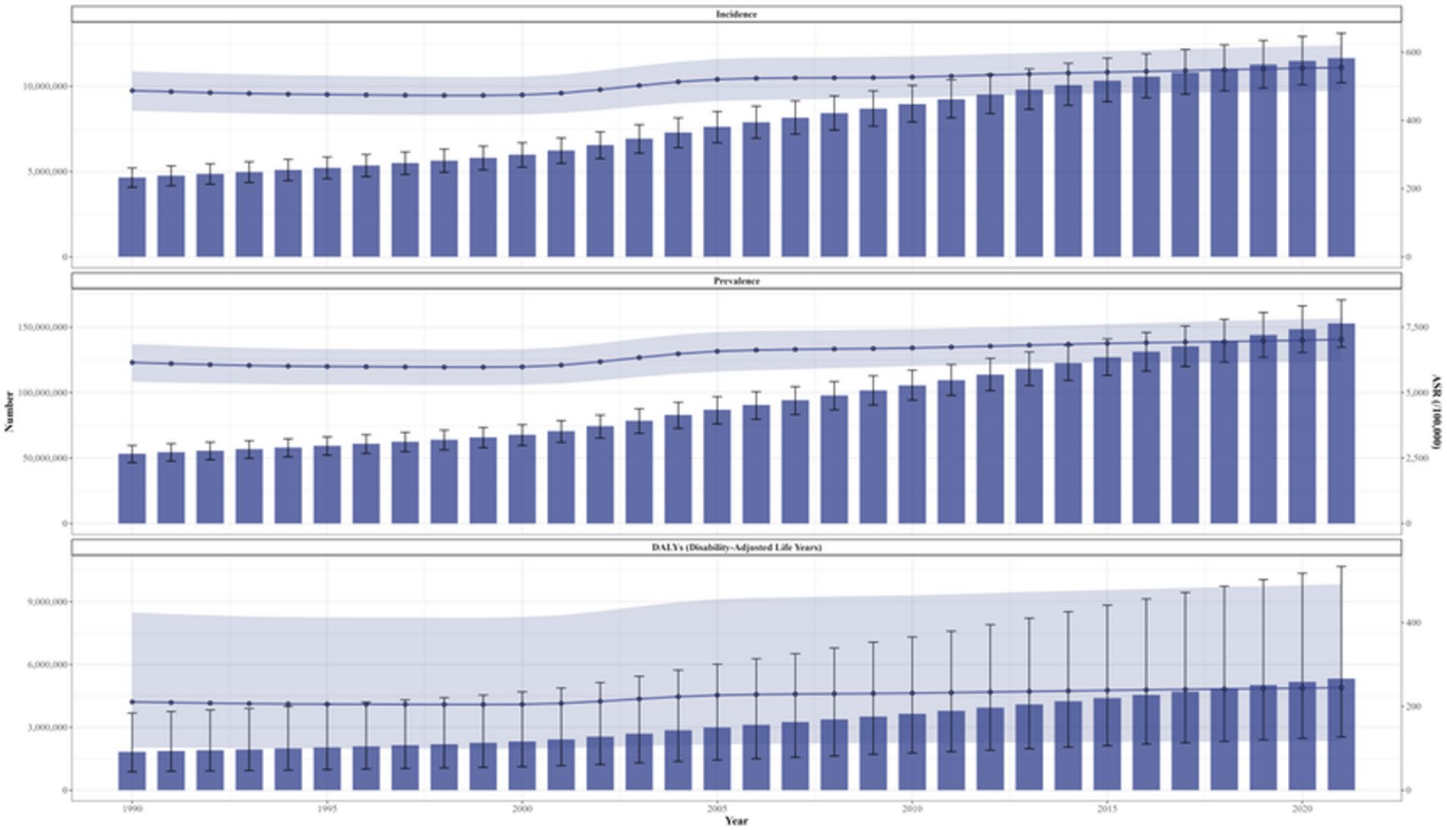
Trends in the changes of ASIR, ASPR, and ASDR for osteoarthritis in China from 1990 to 2021. ASIR, age-standardized incidence rate; ASPR, age-standardized prevalence rate; ASDR, age-standardized disability-adjusted life years rate.

Over the past 32 years, the incidence and prevalence numbers of OA in China increased by 150.37% (95% CI: 150.48–151.64%) and 186.49% (95% CI: 189.36–186.57%). The DALYs increased by 191.19% (95% CI: 188.78–189.79%). The ASRs rose by 13.86% (95% CI: 13.74–14.07%), 14.34% (95% CI: 14.66–14.24%), and 16.23% (95% CI: 15.10–16.06%), respectively (Table 1; Figure 1).

**Figure 2:**
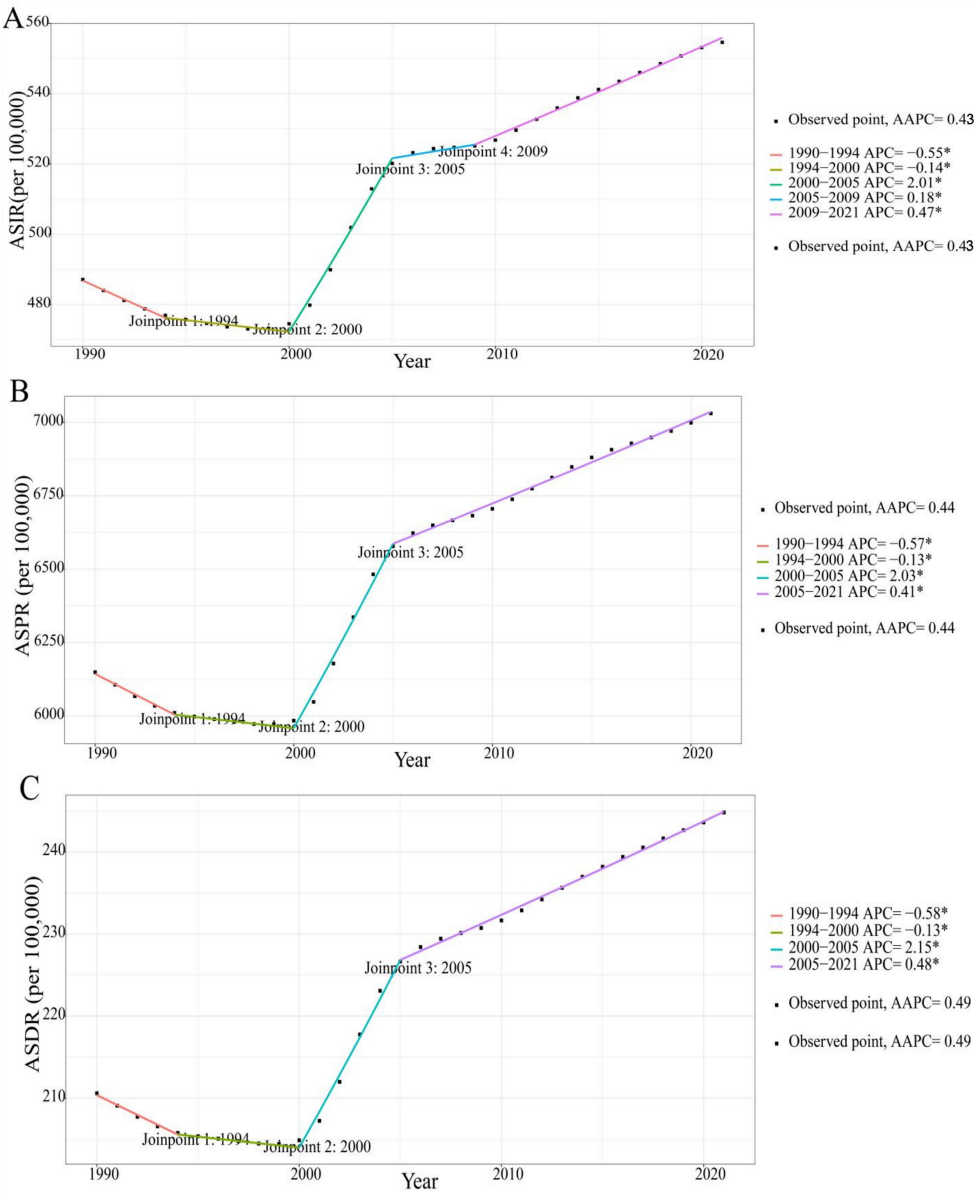
Annual average percentage change of ASIR **(A)**, ASPR **(B)**, and ASDR **(C)** for osteoarthritis in China from 1990 to 2021. ASIR, age-standardized incidence rate; ASPR, age-standardized prevalence rate; ASDR, age-standardized disability-adjusted life years rate.

From 1990 to 2021, the ASRs exhibited varying degrees of growth, with average annual increments of 2.19% (95% CI: 2.12–2.26%), 28.65% (95% CI: 28.06–29.24%), and 1.11% (95% CI: 1.09–1.13%). The lowest points of ASIR and ASPR emerged in 1998, and the lowest point of ASDR occurred in 1999. Additionally, the growth of ASRs was primarily concentrated during 2000–2005, with average annual growth rates of 1.97% (95% CI: 1.85–2.10%), 2.36% (95% CI: 2.23–2.50%), and 2.51% (95% CI: 2.38–2.64%) (Figures 1, 2).

### 3.2 Disease burden of OA across different sexes from 1990 to 2021

**Figure 3:**
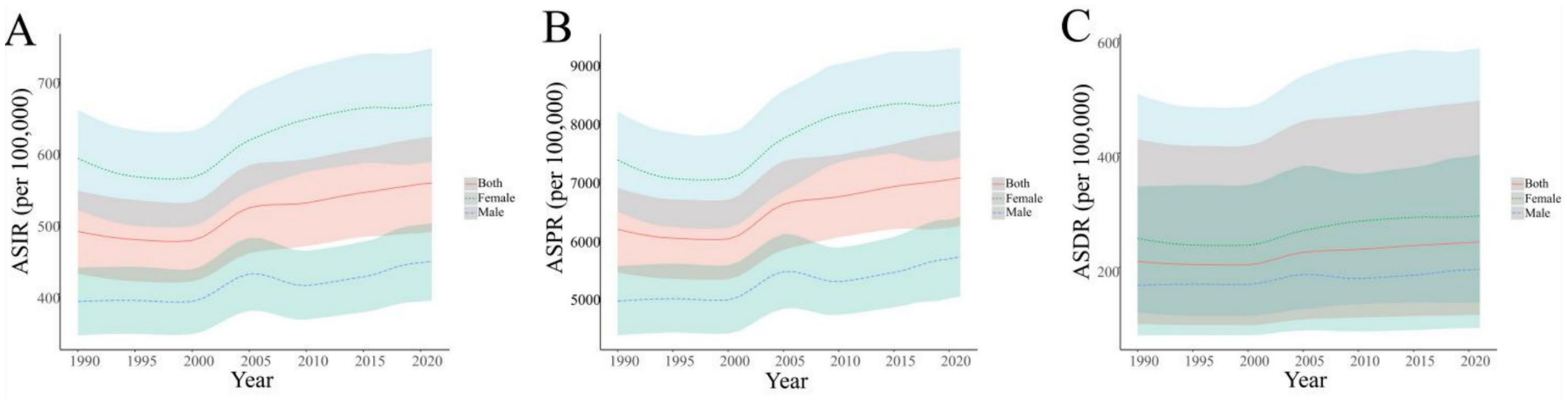
Temporal trends in ASIR **(A)**, ASPR **(B)**, and ASDR **(C)** for osteoarthritis across different sexes in China from 1990 to 2021. ASIR, age-standardized incidence rate; ASPR, age-standardized prevalence rate; ASDR, age-standardized disability-adjusted life years rate.

From 1990 to 2021, the disease burden of OA in China presented obvious sex-related differences. The ASRs of OA were higher among females than among males. Compared with 1990, the ASIR, ASPR, and ASDR among females increased by 12.73, 13.47, and 15.68%, respectively, while among males they increased by 14.42, 15.37, and 16.79%. The increments in ASRs among males were greater than those among females. The change trends of OA among different sexes in China during 1990–2021 were also analyzed. The ASRs of both sexes demonstrated an overall upward trend, and the change processes were largely the same. However, the ASRs among males decreased between 2005 and 2010, causing the ASRs to reach their first peak in 2005 (Figure 3).

### 3.3 Disease burden of OA in different age groups from 1990 to 2021

**Figure 4:**
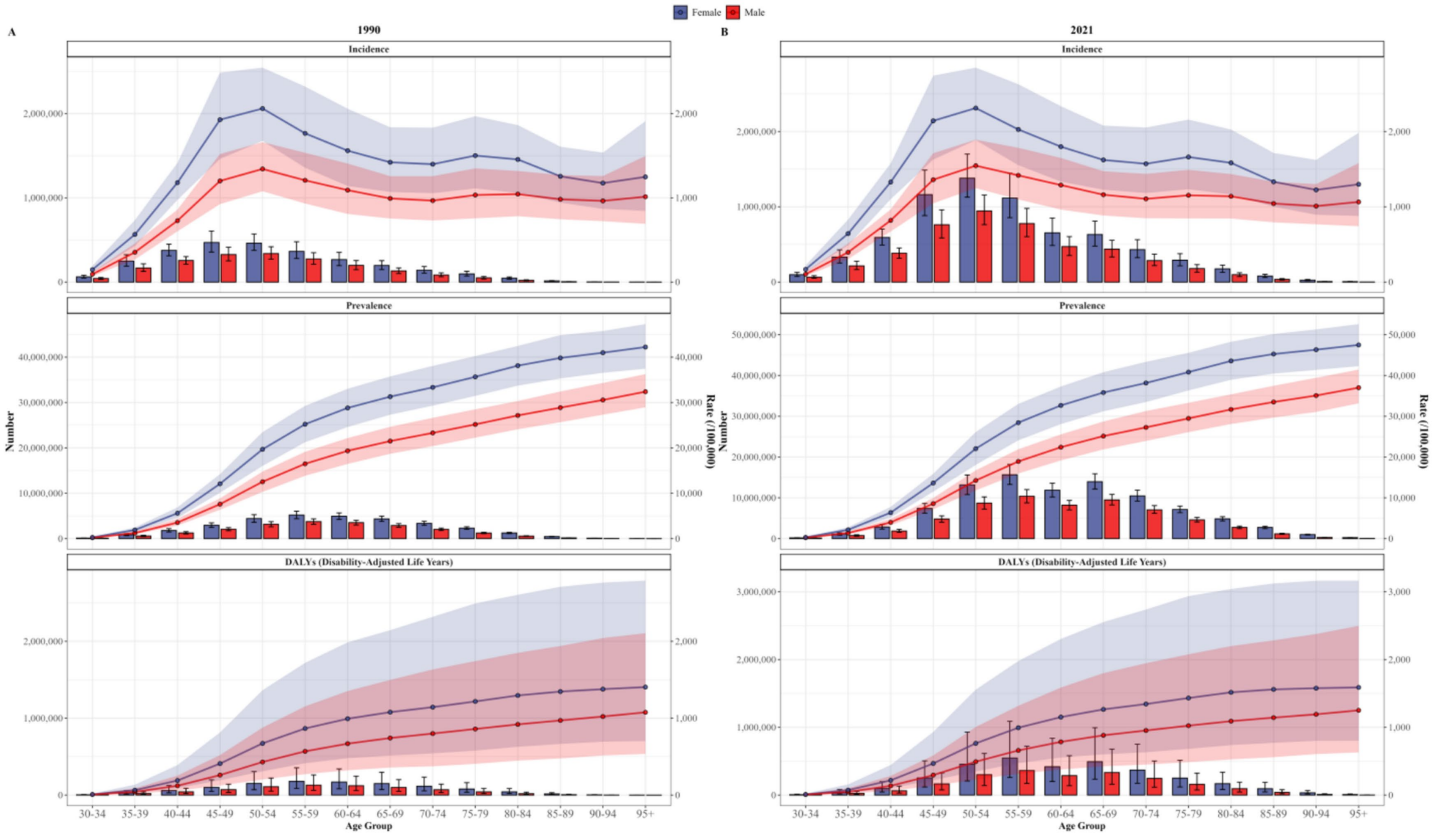
Trends of incidence, prevalence, and DALYs rates for osteoarthritis in different age groups and sexes in China in 1990 **(A)** and 2021 **(B)**.

In 1990 and 2021, the age-distribution of OA among different sexes in China was basically the same. The incidence rates of both males and females reached the highest in the 50–54 age group. The total incidence rate in this age group was 1,924.30 per 100,000 (95% UI: 1,560.47–2,371.26), with 1,546.11 per 100,000 (95% UI: 1,248.30–1,890.58) for males and 2,311.51 per 100,000 (95% UI: 1,888.03–2,847.98) for females. The prevalence rates and DALYs rates of both males and females increased significantly with age, reaching their peaks in the age group of 95 and above. The total prevalence rate in this age group was 47,485.55 per 100,000, and the total DALYs rate was 1,588.88 per 100,000. Among the divided age groups, the incidence, prevalence, and DALYs rates of females were all higher than those of males (Figure 4).

### 3.4 Comparing the changes in the disease burden of OA between China and the world from 1990 to 2021

In 1990, the ASRs of OA globally were higher than those in China. The ASIR of OA worldwide was 489.78 per 100,000 (95% UI: 433.10–541.51), which was higher than 487.11 per 100,000 (95% UI: 428.13–543.75) in China. The ASPR was 6,393.12 per 100,000 (95% UI: 5,683.20–7,059.53), higher than that of China (6,148.92 per 100,000, 95% UI: 5,417.29–6,855.85). The ASDR was 222.80 per 100,000 (95% UI: 106.65–450.29), which was higher than that in China (210.61 per 100,000, 95% UI: 101.91–423.86).

In 2021, the global ASIR, ASPR, and ASDR of OA were 535.00 per 100,000 (95% UI: 472.38–591.97), 6,967.29 per 100,000 (95% UI: 6,180.70–7,686.06), and 244.50 per 100,000 (95% UI: 117.06–493.11), respectively. Compared with China’s 554.61 per 100,000 (95% UI: 486.85–619.54), 7,030.66 per 100,000 (95% UI: 6,211.20–7,831.69), 244.79 per 100,000 (95% UI: 117.30–491.91).

**Figure 5:**
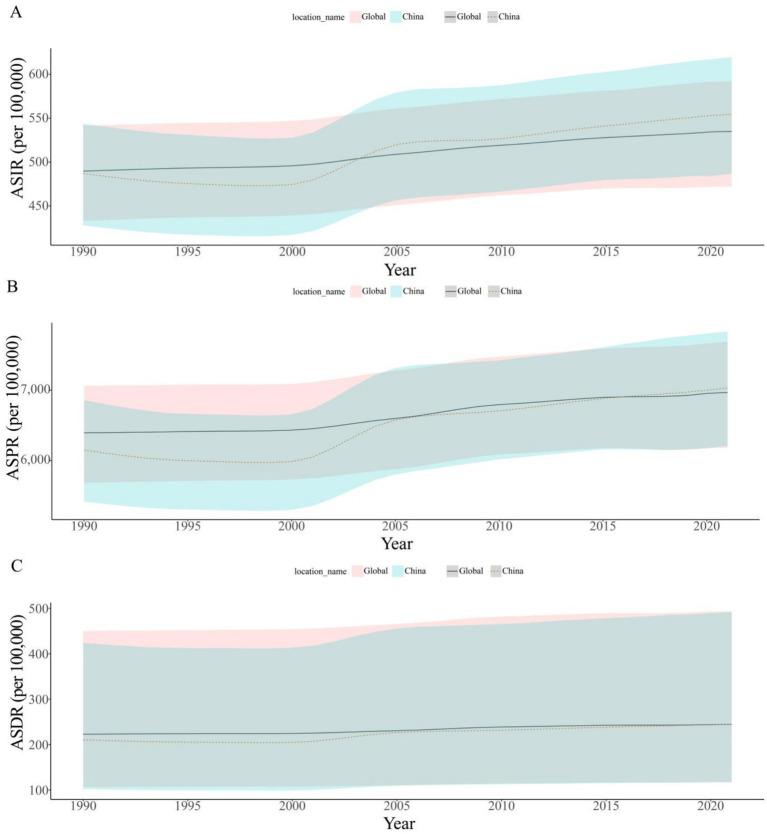
Trends of ASIR **(A)**, ASPR **(B)**, and ASDR **(C)** for osteoarthritis in China and the world from 1990 to 2021. ASIR, age-standardized incidence rate; ASPR, age-standardized prevalence rate; ASDR, age-standardized disability-adjusted life years rate.

From 1990 to 2021, the ASRs of OA in both China and globally generally exhibited an upward trend. The ASDR of global OA became lower than that of China in 2021. Meanwhile, the ASIR and ASPR changed from being higher than those of China to being lower than those of China after 2004 and 2017, respectively (Figure 5).

### 3.5 APC analysis on OA incidence, prevalence, and DALYs rates

**Figure 6:**
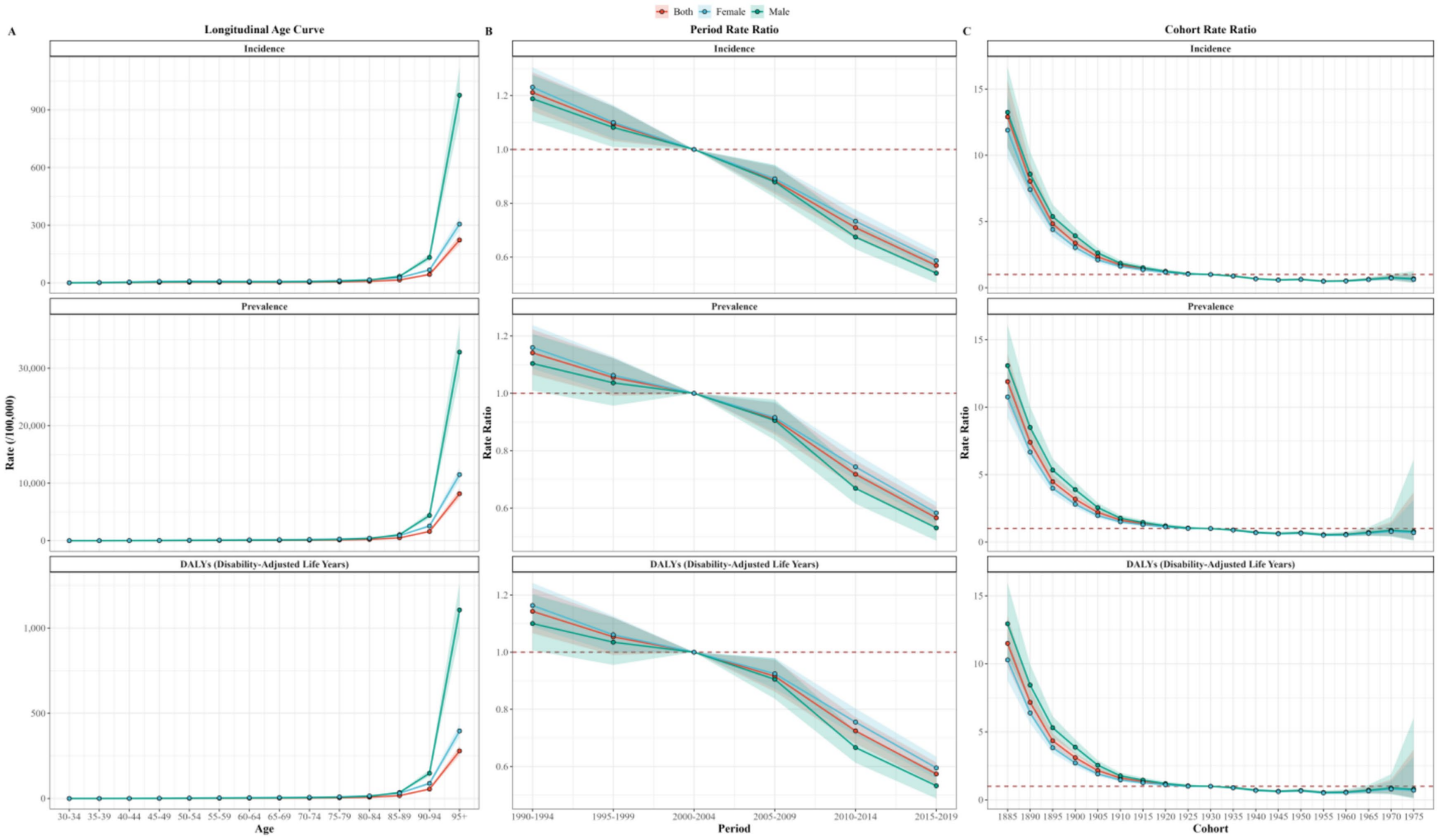
Age **(A)**, period **(B)**, and cohort **(C)** effects for China OA incidence, prevalence, and DALY rate. DALY, disability-adjusted life year; OA, osteoarthritis.

The APC model was used to analyze the age, period, and cohort effects on the incidence, prevalence, and DALYs of OA. Age effect results showed that incidence, prevalence, and DALYs rates increased with age, peaking in the 95 + −y age group, with higher rates observed in men compared to women. Period effect analysis showed that compared to the reference group (2000–2004), the highest Rate Ratios (RRs) for incidence, prevalence, and DALYs were observed during the period of 1990–1994 and the RRs decreased over time. Cohort effect analysis showed a general decline of RRs for incidence, prevalence, and DALYs before the reference group (1930), then remained stable after 1930 (Figure 6).

### 3.6 Decomposition analysis on the DALYs of OA in China from 1990 to 2021

This study evaluated the impact of factors such as aging, population growth, and epidemiological changes on OA epidemiology in China from 1990 to 2021 by performing decomposition analysis on the DALYs of OA.

**Figure 7:**
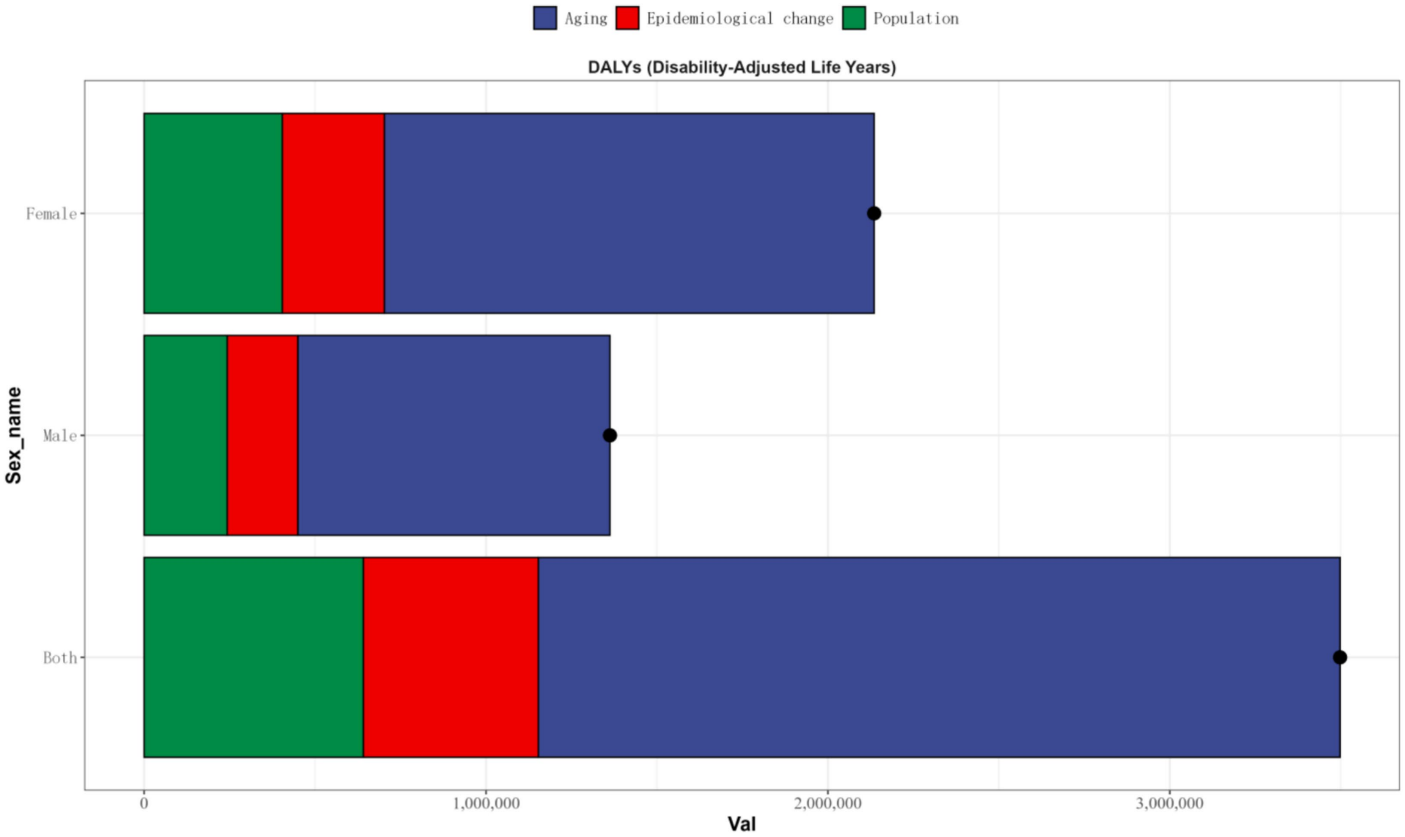
Population-level determinant changes in aging, population growth, and epidemiological changes for OA DALYs in China from 1990 to 2021. Black dots represent the total change contributed by all three components. A positive value for each component indicates a corresponding positive contribution in DALYs, and a negative value indicates a corresponding negative contribution in DALYs. DALY, disability-adjusted life year; OA, osteoarthritis.

Aging, population growth, and epidemiological changes were driving an increase in the disease burden of osteoarthritis in China. Aging, population growth, and epidemiological changes contributed 67.04, 18.34, and 14.63%, respectively, to the rise in disease burden for both genders (Figure 7).

### 3.7 Adult mean age-standardized prevalence contributing to OA in China

**Figure 8:**
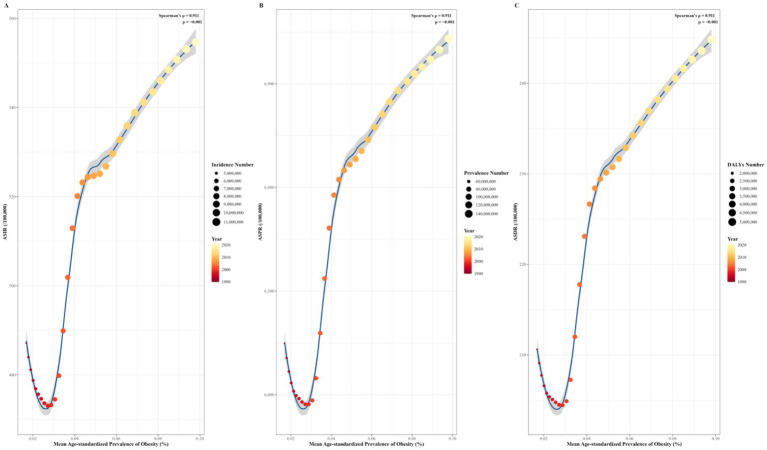
Correlation between ASIR **(A)**, ASPR, **(B)** and ASDR **(C)** of OA and adult mean age-standardized prevalence in China from 1990 to 2021. ASIR, age-standardized incidence rate; ASPR, age-standardized prevalence rate; ASDR, age-standardized disability-adjusted life years rate.

From 1990 to 2021, the adult mean Age-Standardized prevalence in China increased from 1.68 to 9.8%. Significant positive correlations between the ASRs (including ASIR, ASPR, and ASDR) and the adult mean Age-Standardized prevalence from 1990 to 2021 were observed after 1994, with ASIR (r = 0.911, *p* < 0.001), ASPR (r = 0.911, *p* < 0.001), and ASDR (r = 0.911, *p* < 0.001). Conversely, significant negative correlations were observed before 1994 (Figure 8).

### 3.8 Prediction of disease burden of OA in China from 2022 to 2050

In order to gain an in-depth understanding of the trend of the OA disease burden in China after 2021, the BAPC model was adopted to forecast the number of incidence, prevalence, and DALYs as well as ASRs of OA during the period from 2022 to 2050. The prediction results revealed that subsequent to 2021, the ASIR of OA in China would ascend from 554.61 per 100,000 (95% UI: 486.85–619.54) in 2021 to 556.755 per 100,000 (95% UI: 458.23–655.27) in 2050. The number of new cases would be 7,093,339.96 (95% UI: 5,837,879.84–8,348,800.07). The ASPR of OA in China would increase from 7,030.66 per 100,000 (95% UI: 6,211.20–7,831.69) in 2021 to 7,762.05 per 100,000 (95% UI: 6,748.24–8,775.86) in 2050. The number of patients would be 988,997,047.69 (95% UI: 85,979,958.16–111,811,4137.23). The ASDR of OA in China would increase from 244.79 per 100,000 (95% UI: 117.30–491.91) in 2021 to 269.98 per 100,000 (95% UI: 232.27–307.69). DALYs would reach 3,439,909.71 person—years (95% UI: 2,959,493.10–3,920,326.32).

**Figure 9:**
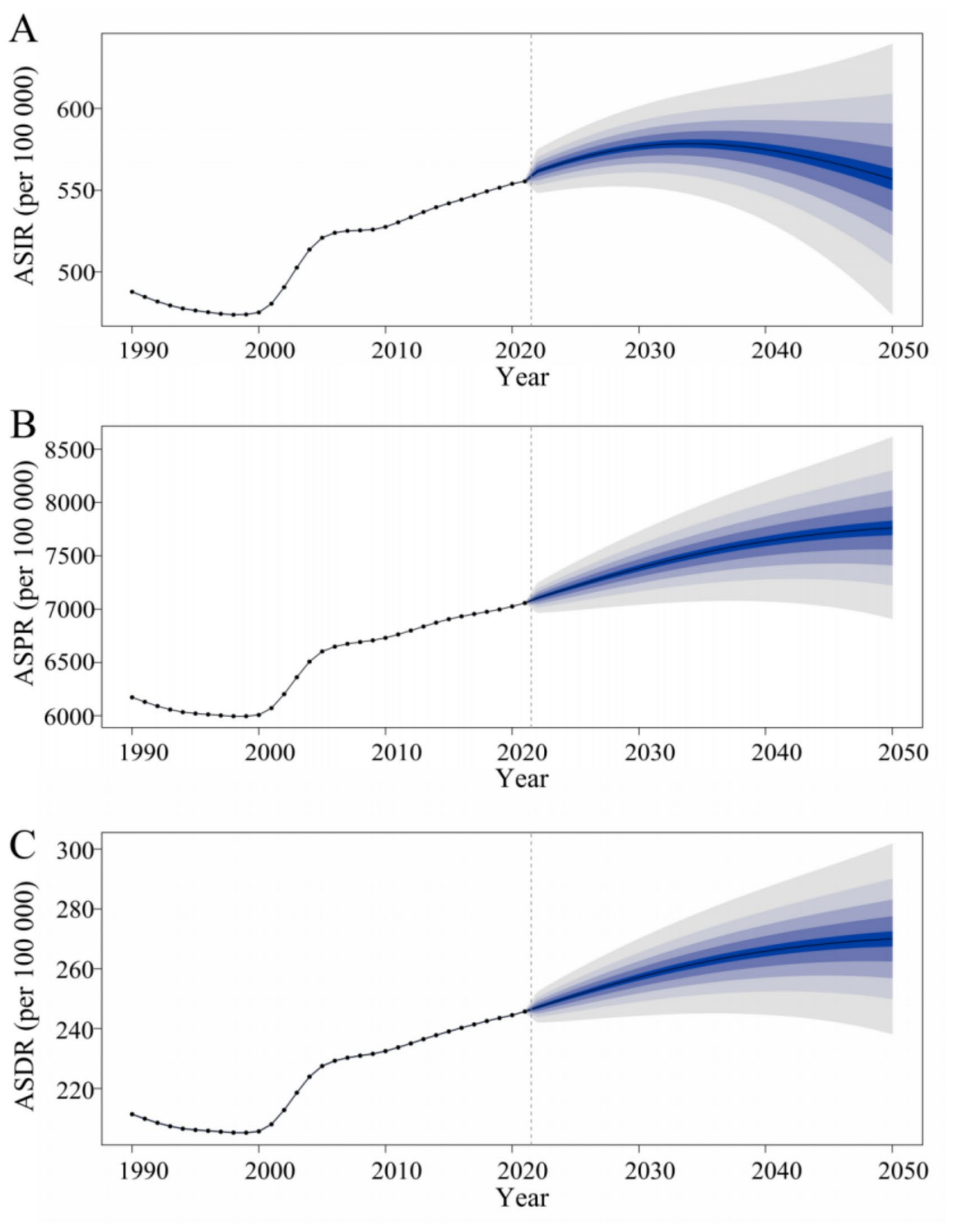
Predicted trends in ASIR **(A)**, ASPR, **(B)** and ASDR **(C)** for osteoarthritis in China from 2022 to 2050. ASIR, age-standardized incidence rate; ASPR, age-standardized prevalence rate; ASDR, age-standardized disability-adjusted life years rate.

From 2022 to 2050, the ASIR of OA in China would first increase and then decrease, with a maximum value of 578.52 per 100,000 (95% UI: 541.97–615.07) in 2034. Both ASPR and ASDR would exhibit an upward trend, yet the rate of increase would gradually decline. It is thus likely that the burden of OA in China will continue to grow in the future (Figure 9).

## 4 Discussion

This study has revealed that the disease burden of OA in China was on the rise during the period from 1990 to 2021. The ASRs, respectively, witnessed an increase of 13.86% (95% UI: 13.74–14.07%), 14.34% (95% UI: 14.66–14.24%), and 16.23% (95% UI: 15.10–16.06%). By 2021, the number of OA patients in China had reached 152,848,105.93 (95% UI: 134,655,962.16–170,842,262.84), accounting for approximately a quarter of the global patient number. Consequently, the prevention and treatment of OA in China require strengthening.

The analysis of the alteration in the disease burden of OA in China from 1990 to 2021 indicated that the trend of change in ASRs of OA in China first decreased and then increased. The lowest points of ASIR and ASPR were in 1998, and the lowest point of ASDR was in 1999, with its growth mainly concentrated during 2000–2005. The reason for the decline in the disease burden of OA is that the public health reform in China during this period led to an improvement in the medical level, enabling more patients to diagnose and treat OA at an early stage (33).

With the development of China’s social and economic level, people’s nutrient intake has become more comprehensive. Moreover, with the implementation of health—promotion policies, people have become aware of the significance of physical exercise. This has positive implications for preventing the occurrence of OA and reducing the disease burden.

In contrast, the growth of the disease burden of OA is associated with the increasing aging of the population (34), the rise of obesity (35), the increase of diagnoses (36), and the increase of sports injuries (37). Several studies have demonstrated that obesity is the most crucial risk factor for the occurrence and progression of OA (38). The increasing rate of age-standardized summary exposure value of high body mass index in China has been more than twice the global average level since 2010. The increments in deaths and DALYs attributable to high body mass index in China ranked 59th and 52nd among 204 countries/territories worldwide in the past 30 years (39). Spearman’s correlation analysis revealed significant positive correlations between the ASRs and the mean Age-Standardized prevalence from 1994 to 2021. Existing studies have demonstrated that immune mechanisms play a crucial role in the pathogenesis of obesity and osteoarthritis (40). Account for the aforementioned factors, the increase in the ASRs of OA in China is closely linked to the rise in obesity rates.

This study has revealed that the disease burden of OA in China exhibited obvious sex differences. From 1990 to 2021, the ASRs of OA in China were higher among females than among males. However, during this period, the growth rate of ASRs in males was higher than that in females. The proportion of females in the total OA population in China rose from 60.09% in 1990 to 60.59% in 2021. The higher disease burden of OA in females compared to males might be associated with the unique cartilage degradation pattern in females (41), the significant regulatory effect of estrogen on bone growth, metabolism, and tissue homeostasis (42), the higher risk of lateral fascial compartment disease in females due to differences in physiological structure (43), and the higher incidence of traumatic fractures in females resulting from weak joint support.

Age is one of the significant influencing factors of OA. In all age groups within this study, the incidence among both males and females reached its peak within the 50–54—year—old age group. The incidence rate was 1,546.11 per 100,000 (95% UI: 1,248.30–1,890.58) for males and 2,311.51 per 100,000 (95% UI: 1,888.03 – 2,847.98) for females. Nevertheless, both the prevalence and DALYs rates rise with age. The possible reason for the highest incidence in the 50–54—year—old age group might be that bone growth and metabolism are readily influenced by hormone levels, and changes in hormone levels such as estrogen during menopause are associated with other factors (44). The increase in prevalence with age is related to the scarcity of effective treatments for OA and the aging population (2).

When comparing the disease burden of OA between the world and China during the period from 1990 to 2019, in 1990, the number of OA patients in China accounted for merely one—fifth of the total number of patients worldwide. However, in 2021, this proportion has reached one—fourth. While the disease burden of OA in China is steadily increasing, the proportion of OA in the world is also on the rise. From 1990 to 2021, the disease burden of OA in both the world and China grew over time, yet the growth rate of the global disease burden was lower than that of China. In 2021, the ASDR of global OA shifted from being higher than that of China to being lower, while the ASIR and ASPR of global OA changed from being higher than those of China to being lower after 2004 and 2017, respectively. It has been demonstrated that the disease burden of OA is associated with income level and Socio-Demographic Index (SDI) (45). OA is one of the most prevalent diseases in high—income countries. In recent years, with the continuous development of China’s social and economic level, the disease burden of OA has increased along with the rise in China’s per capita income. The highest prevalence was found in high—middle SDI countries, and the highest incidence and DALYs were identified in middle SDI countries (46), which is also in line with China’s SDI. Additionally, among 204 countries and regions, China’s ASIR and ASPR were higher than expected, while the ASDR was lower than expected. This also reflects the continuous improvement in the treatment of OA in China.

According to the BAPC prediction results, from 2022 to 2050, the ASIR of OA in China first increased and then decreased, reaching a maximum of 578.52 per 100,000 (95% UI: 541.97–615.07) in 2034. Both the ASPR and ASDR exhibited an upward trend, yet the rate of increase was gradually decreasing. These may be related to China’s family planning policy. In the 1970s, faced with high birth rates, the Chinese government became increasingly concerned about the strain on resources caused by the rapidly growing population. As a response, starting from 1973, the government began promoting the ‘later-longer-fewer’ policy to couples, which encouraged delayed marriage and childbearing, longer spacing between births, and having fewer children (47). The one-child policy was officially launched in 1978, and ‘family planning’ was first included in the Constitution, since then, the population policy characterized by ‘late marriage, long intervals between births, and fewer children’ has basically taken shape and continued for 35 years (48, 49). In 2007, Chinese authorities claimed the policy had helped prevent 400 million births due to the policy (50). We speculate that when this portion of the population, which has a smaller base, reaches the age at which OA incidence peaks due to being born during the ‘family planning’ policy era, the ASIR will passively decline in sync with the population size, resulting in a ‘post-peak decline’ phenomenon on the predictive curve. Furthermore, this explains why the growth slope of China’s ASPR and ASDR is gradually decreasing in the future prediction.

Most current studies on risk factors for OA development focus on the progression of OA disease, specifically pathological changes in joint tissues. However, there is limited evidence regarding risk factors for OA symptoms and complaints. This lack of evidence makes it difficult to identify at-risk populations for osteoarthritis and implement effective interventions, and compliance among high-risk populations is also one of the major challenges in preventing osteoarthritis (51). As China’s population ages, research predicts that there will be 14.02 million older adults people in need of long-term care in China by 2030 (52). Given the significant impact of advanced age on the risk factors for OA and the irreversibility of age, preventive efforts for the older adults should focus more on tertiary prevention to reduce disability and early screening for middle-aged individuals might be more meaningful and valuable (1, 53). In summary, paying attention to controllable factors such as individual complaints and symptoms, and providing them with motivation to persist in preventive measures is particularly important in the prevention and treatment of OA. We recommend establishing a regular screening system for osteoarthritis among high-risk populations to identify high-risk individuals early, allowing timely intervention and management. The screening should integrate clinical characteristics and relevant indicators to assess the progression of osteoarthritis, ensuring that patients receive timely treatment. Based on the characteristics of different regions in China, formulate corresponding health management plans and provide personalized preventive and treatment recommendations. Preventing osteoarthritis is a comprehensive task that requires the concerted efforts of society as a whole.

This paper has systematically analyzed the disease burden of OA in China based on the GBD 2021 database. However, there are still several limitations. For instance, the GBD2021 database consists of not real—life data but rather indicators computed through mathematical modeling based on data collected from various monitoring systems or surveys. This also gives rise to the possibility of selection bias within the collected data itself, resulting in errors when compared with the real—world data. Additionally, the GBD2021 database is not the outcome of large—scale survey statistics following rigorous design for OA, and some OA-related indicators might have been overlooked. Due to the lack of provincial—level OA disease data in China, the results of the OA disease burden analysis in this paper do not represent the disease burden of OA in each individual province.

## 5 Conclusion

In conclusion, this study demonstrated that the disease burden of OA in China was on the rise and surpassed the global average during the period from 1990 to 2021. In China, the prevalence and DALYs rate of OA gradually increased with age, and the incidence peaked in the 50–54 age group. The disease burden among females was higher than that among males. In recent years, the increase in China’s OA ASR has been closely related to the rise in obesity rates. Consequently, more attention should be focused on middle-aged and older adults females in the future, as this is also the key to the prevention and treatment of OA in the future.

## Data Availability

The original contributions presented in the study are included in the article/supplementary material, further inquiries can be directed to the corresponding author.

## Footnotes

^1^ http://ghdx.healthdata.org/
